# Fracturing Behaviors and Mechanism of Serial Coal Pillar Specimens with Different Strength

**DOI:** 10.3390/ma16072690

**Published:** 2023-03-28

**Authors:** Cheng Song, Guangming Cao, Jinwen Bai, Shanyong Wang, Guorui Feng, Xudong Shi, Kai Wang, Chun Zhu

**Affiliations:** 1 College of Mining Engineering, Taiyuan University of Technology, Taiyuan 030024, China; 2 Key Laboratory of Shanxi Province for Mine Rock Strata Control and Disaster Prevention, Taiyuan University of Technology, Taiyuan 030024, China; 3 Postdoctoral Workstation, Shanxi Research Institute of Clean Energy, Tsinghua University, Taiyuan 030032, China; 4 Priority Research Centre for Geotechnical Science & Engineering, The University of Newcastle, Callaghan, NSW 2308, Australia; 5 School of Earth Sciences and Engineering, Hohai University, Nanjing 210098, China

**Keywords:** fracturing behaviors, unbalanced deformation characteristics, key deformation element, progressive fracturing mechanism, serial coal pillar specimens

## Abstract

The fracturing behaviors of serial coal pillars is significant for understanding their failure mechanism. To reveal this, the bearing stress, acoustic emission, electrical resistivity, local strain, force chain distribution, and cracks evolution of serial coal pillars under uniaxial compression were evaluated by experiment and numerical simulation. The results show that four bearing stages are observed during the fracturing process (i.e., nonlinear growth, linear growth, yielding growth, and weakening stages). The acoustic emission features, electrical resistivity responses, strain develops, force chain distributions, cracks evolutions, and local displacement are highly consistent to illustrate the fracturing behaviors. System fracturing of serial coal pillar specimens is appeared along with the collapse of lower uniaxial compressive strength coal pillar specimen. The limit bearing capacity of serial coal pillar specimens is almost equal to the strength of lower uniaxial compressive strength coal pillar specimen. The unbalanced deformation characteristics of serial coal pillar specimens are presented due to the strength differences. The evolution of the key deformation element is the rooted reason for the overall fracturing mechanism of serial coal pillar specimens. For serial coal pillar specimens with different strengths, the critical condition of system fracturing is that the sum of secant modulus of upper and bottom coal pillars is zero, which is expected to predict the system fracturing of serial pillars in the underground coal mining.

## 1. Introduction

In recent years, an increasing number of ultra-closed mining activities have been conducted in China [1,2], America [3,4], Australia [5,6], India [7], and other famous coal-mining countries [8,9]. According to different mining methods at the upper and bottom levels, various multi-seam mining configurations are defined. Multi-seam pillar mining is one of the main configurations in practice [10]. Generally, several residual coal pillars are arranged and left permanently in ultra-closed mining to support the weight of overburden strata, which are overlapped or staggered at the different levels [11,12]. Overlapped residual coal pillars can be considered as a whole system [13], which can also be termed as serial coal pillars.

Long-term stability of serial coal pillars is one of the significant issues to be focused on in the ultra-closed goaf [14]. With the coupled effects of environmental weathering, disturbance loads, and goaf fires, the strength of serial coal pillars is gradually deteriorated [15,16,17]. System stability of serial coal pillars is also weakened progressively. As a result, catastrophic collapse of the upper or bottom coal pillars can be induced. Instability damage of serial coal pillars is usually powerful, destructive, and dangerous [6,18,19]. Therefore, it is particularly necessary to investigate the fracturing behaviors of serial coal pillars in the ultra-closed goafs.

Tremendous efforts and attempts have been undertaken to study the fracturing behaviors of serial coal/rock masses. In view of theoretical research, Chen et al. [20] obtained the theoretical micro seismic event rate generated by the serial rock specimens under uniaxial compression. A laboratory test with the same setup was also conducted to verify the theoretical analysis while the related experimental results were limited in the serial granite and marble specimens. Wang et al. [21] proposed a cusp-type catastrophe model of rock-rock specimens and studied the instability mechanism. The theoretical analysis showed that the stability characteristic of the rock–rock system was determined by the stiffness ration. Nevertheless, no further experimental tests were conducted to examine the above conclusions. Zhao et al. [22] derived a compression-shear fracturing criterion of composite coal–rock body considering the interface effect. Accuracy of the analytical model was also verified by the triaxial compressive tests. However, the loading conditions of composite structures and the serial coal pillars were different [14,21,23]. The obtained model could not be used directly to explain the progressive fracturing mechanism of serial coal pillars.

In addition, several numerical programs have been applied to investigate the fracturing behaviors of serial coal/rock masses. For example, Wang et al. [10] built up a Fast Lagrangian Analysis of Continua (FLAC) model to investigate the principal stresses, yield zones and the stability of overlapped gypsum pillars. Singh et al. [7] ran several FLAC^2D^ and FLAC^3D^ models to obtain the safety factor contours of serial coal pillars and the parting. Porathur et al. [24] analyzed the stress-strain curves and plasticity states of staggered coal pillars based on the FLAC^3D^ models. Al Heib et al. [5] investigated the plasticity and shear strain development of serial chalk pillars by the FLAC^3D^ numerical modeling method. Wang et al. [14,21] applied the Rock Failure Process Analysis code (RFPA^2D^) to simulate the damage accumulation, seismic energy release and failure characteristics of serial rock-rock mass during the uniaxial loading process, which confirmed that the failure of serial rock-rock system was a progressive process. Furthermore, Liakas et al. [25] and Behnia et al. [26] captured the crack propagation and determined the strength of serial rock mass with the particle flow code (PFC). The associated numerical results were compared with typical case studies, while they were still not fully validated by field data or laboratory results. Compared with the field monitoring method, the laboratory testing is relatively repeatable and easy-operating. It is also convenient for achieving different loading conditions and adjusting physical parameters of specimens. Therefore, laboratory testing is needed to be performed to make sufficient verification concerning the numerical modeling.

From the laboratory perspective, several tests have been conducted to illustrate the fracturing behaviors and mechanism of serial coal/rock masses. Greco et al. [27,28] utilized various kinds of serial rock specimens in the uniaxial compressive tests to determine the overall mechanical parameters. The results showed that the compressive strength, elastic modulus, and Poisson’s ratio of serial rock specimens were different from those of single component. The fracturing mechanism of serial rock specimens was also different from that of single component. For the former one, the failure depended on the properties of upper and bottom coal specimens. The overall collapse was induced by the interaction of the two independent specimens in the serial system. For the later one, the failure was controlled by the properties of single rock specimen. The instability was induced by the damage of defect area in the single rock component. Mohamed et al. [29] carried out the uniaxial compressive experiments to determine the influence of weak rock thickness and moisture content on the strength of serial sandstone-shale specimens. Tziallas [30] proposed a methodology to predict the strength of serial sandstone and siltstone specimens based on the laboratory results. Berisavljević et al. [31] inspected the influence of lithological heterogeneity on serial sandstone-siltstone specimens’ strength. Furthermore, Lu et al. [32], Huang et al. [33], and Liu et al. [34] investigated the mechanical behavior and failure mode of serial coal-rock specimens by a group of uniaxial compression tests.

These laboratory tests provided a deeper understanding about the fracturing of serial coal/rock masses. However, all of the experimental tests were just concentrated on the serial rock-rock specimens or serial coal-rock specimens. Due to the differences of combining size and contacting form, the above laboratory results could not be directly applied in the serial coal-coal specimen testing. The problem to be solved was also different. The serial rock-rock specimens were prepared to reveal the mechanism of rock burst. The serial coal-rock specimens were proposed to investigate the interaction of roof, coal seam and floor at the single mining level. However, the serial coal-coal specimens were designed to explore the interaction of upper and bottom coal pillars at the ultra-closed goafs. Therefore, further laboratory observations about serial coal pillar specimens with different mechanical properties are necessary to make a throughout insight about the fracturing behaviors and mechanism. Meanwhile, it should also be verified by corresponding numerical simulation and explained by the related theorical method.

**Figure 1:**
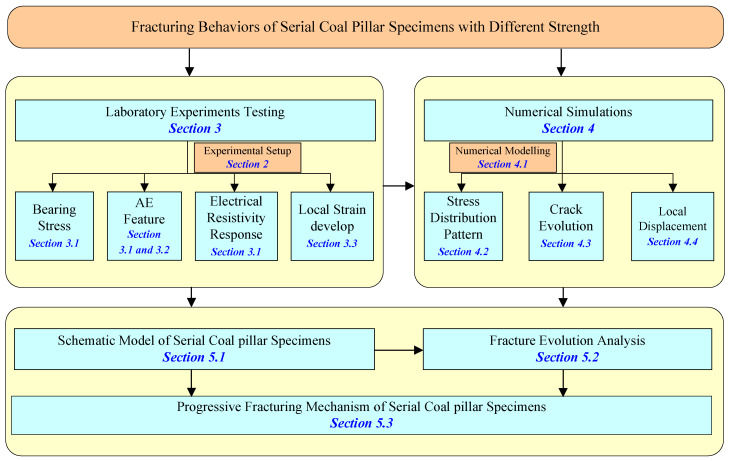
Overview of research flowchart.

In this study, the laboratory uniaxial compression tests and numerical simulations are conducted for serial coal pillar specimens with different strength. During the loading process, the fracturing behaviors are investigated comprehensively based on bearing stress, acoustic emission (AE) features, electrical resistivity responses, strain develops, force chain distributions, cracks evolutions, and local displacement. A schematic model of serial coal pillar specimens is also established. Then, the key deformation element is defined and the fracturing evolution is analyzed to reveal the unbalanced deformation characteristics. Finally, the progressive fracturing mechanism is revealed theoretically. Figure 1 shows a flowchart summarizing the main content of this research.

## 2. Experimental Test Setup

In this section, the experimental specimens, experimental testing system, and experimental scheme will be introduced in detail.

### 2.1. Experimental Specimens

The serial coal pillars were generally distributed at different goaf levels. They played a combining bearing role with the surrounding rock strata [6,10,35]. That is, the roof strata, upper coal pillar, parting strata, bottom coal pillar, and floor strata should be considered as a whole system. Previous researches had confirmed that the coal pillars were easier to be damaged than the rock strata in the combining bearing system. That is, the stability of serial coal pillars should be paid much more attention [36]. Once the initial fracturing of serial coal pillars was controlled with proper measurements, the stability of the whole bearing system could then be maintained. Therefore, two overlapped cylindrical specimens were designed to model the serial coal pillars without consideration of parting rock in this study.

**Figure 2:**
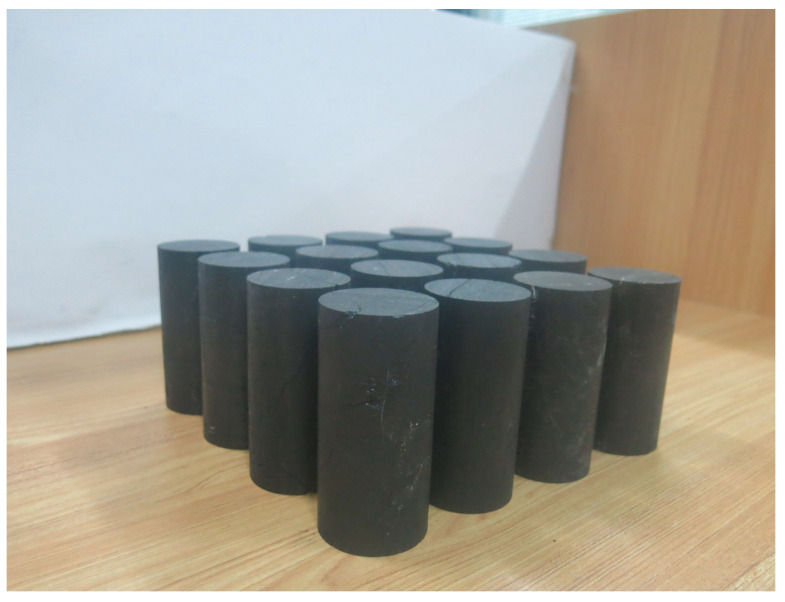
Experimental coal pillar specimens.

Obtained from the Datong coal basins of Shanxi in China, the experimental coal pillar specimens were prepared from natural coal mass. According to the methods suggested by the International Society for Rock Mechanics [37], the coal pillar specimens were cored, shaped and polished carefully. The diameter of experimental coal specimens was 50 mm, while the height was 100 mm. Afterwards, the polished coal pillar specimens were kept in a constant temperature and humidity incubator for 24 h before the experiment. The temperature and humidity were set to 20° and 70%, respectively. Figure 2 shows the photograph of experimental coal pillar specimens.

Two types of coal pillar specimens were prepared in this experiment, which were collected from different mining level in Datong coal field. Table 1 lists the basic physical and mechanical parameters of testing coal pillar specimens, which are determined averagely from five series of tests.

### 2.2. Experimental Testing System

**Figure 3:**
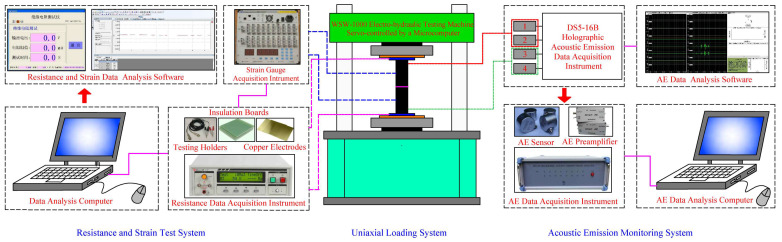
Experimental system.

The experimental testing system consisted of uniaxial loading, AE monitoring, electrical resistance testing, and strain testing systems, as shown in Figure 3. During the fracturing process of specimens, the mechanical behaviors, AE features, electrical resistivity responses and local strain develops could be monitored simultaneously by this testing system.

#### 2.2.1. Uniaxial Loading System

In the present experiments, the confining stress of serial coal pillar specimens was regarded to be very small since the adjacent coal resources were excavated. Furthermore, the axial stress of serial coal specimen with a deeper buried condition was relatively large. Therefore, the axial stress was viewed as the main loading factor associated to the fracturing of serial coal pillar specimens. The axial loading condition was thus selected in the following laboratory experiments.

**Figure 4:**
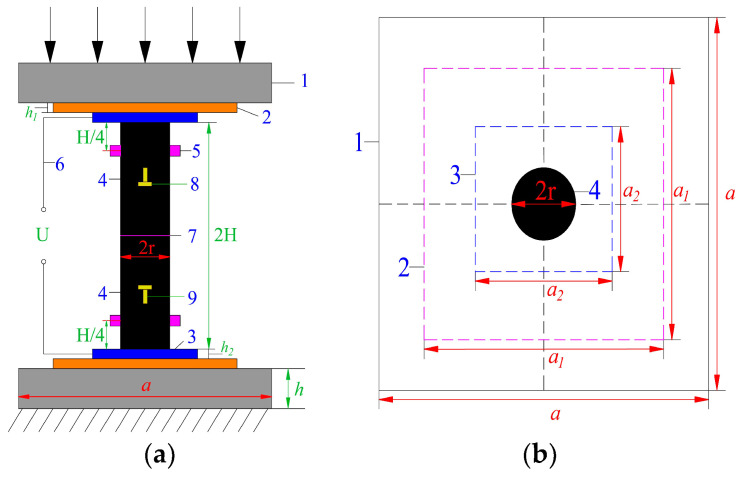
Schematic diagram of serial coal pillars model in the laboratory test. (**a**) Lateral view; (**b**) Top view. Note: 1—loading plates; 2—insulation boards; 3—copper electrodes; 4—coal pillar specimens; 5—AE sensors; 6—resistance testing holders; 7—interface of coal pillar specimens; 8—horizontal strain gauge; 9—vertical strain gauge; *H*—height of single coal pillar specimen; *r*—radius of coal pillar specimen; *a*—length and width of loading plate; *h*—height of loading plate; *a*_1_—length and width of insulation board; *a*_2_—length and width of copper electrode; *U*—Voltage of insulation resistance testing system; *h*_1_—height of insulation board; *h*_2_—height of copper electrode.

A servo-controlled testing machine was applied to achieve the uniaxial loading condition. As shown in Figure 3 and Figure 4, two rigid steel loading plates, with dimensions of a × a × h = 400 mm × 400 mm × 80 mm, were placed between the loading frame and the testing coal specimens.

In this study, the displacement control model was selected according to similar papers related to the fracturing of coal/rock pillars [14,21,23]. Each displacement growth meant the mining disturbance to the serial coal pillar specimens [14,38]. Along with the growth of axial displacement, the stress state at the defect areas in the serial coal pillar specimens was satisfied to the strength criterion. Then, the damage would be induced at such areas to reach a new equilibrium state. At this situation, the stress redistribution was induced, which would give birth to further ruptures of adjacent areas in the serial coal pillar specimens [14]. That is, the stress redistribution was constantly repeated during the loading process. It would finally lead to the macroscopic fracturing of serial coal pillar specimens. Therefore, the displacement rate was 0.12 mm/min in the control model.

The upper loading plate was stationary during the loading process, while the bottom loading plate was movable. The displacement boundary conditions were shown as follows: (1) the axial displacement was zero at the upper boundary of serial coal pillar specimens; (2) for the bottom boundary, the axial displacement was equal to the rising distance of bottom loading plate. It was the product of displacement-control rate (0.12 mm/min) and the loading time. Besides, the stress boundary conditions were also presented: the horizontal stress was zero at the upper and bottom boundaries of serial coal pillar specimens since the end frictional effect in this testing was neglected [39].

#### 2.2.2. AE Monitoring System

Real-time AE features can make a clear explanation about the internal damage of testing materials [40,41]. The AE monitoring system was utilized to observe the AE characteristics of serial coal pillar specimens. As shown in Figure 3, it consisted of AE sensors, AE preamplifiers, an AE data acquisition instrument and data processing system.

Four sensors (RS2A-1, RS2A-2, RS2A-5, RS2A-6) were employed to record the AE parameters. Two sensors (RS2A-2, RS2A-6) were attached on the surface of upper coal pillar specimen. The other ones (RS2A-1, RS2A-5) were located on the surface of bottom coal pillar specimen. The arrangement position of AE sensors is shown symmetrically in Figure 4. To improve the signal transmission, silicon grease was applied as coupling agent between AE sensors and coal pillar specimens. The data acquisition frequency and amplitude threshold were set at values of 2.5 MHz and 40 dB, respectively.

#### 2.2.3. Electrical Resistance Testing System

Electrical resistivity is an inherent physical property that defines the resistance characteristics of testing specimen [42,43]. The responses of electrical resistivity can reveal the fracturing behaviors of coal/rock at different loading stages [44] which are helpful for predicting the dynamic collapse induced by pillars’ instability. In the current experiments, serial coal pillar specimens as a whole were regarded as an electrical resistance element.

Generally, the electrical resistivity of a single coal pillar specimen is determined [45] by Equation (1).

(1) $$\rho =R\cdot \frac{S}{H}=R\cdot \frac{\pi r^{2}}{H}$$

where *ρ* is the electrical resistivity of a single coal pillar specimen (Ω·m); *R* is the electrical resistance of a single coal pillar specimen (Ω); *S* is the effective cross-sectional area of a single coal pillar specimen (m^2^); *H* is the height of a single coal pillar specimen (m); *r* is the radius of a single coal pillar specimen (m)

For the serial coal pillar specimens, the electrical resistivity can be calculated by the Equation (2).

(2) $$\rho_{p}=R_{p}\cdot \frac{S_{p}}{H_{p}}=(R_{u}+R_{b})\cdot \frac{S_{p}}{2H}=(R_{u}+R_{b})\cdot \frac{\pi r^{2}}{2H}$$

where *ρ_p_* is the electrical resistivity of serial coal pillar specimens (Ω·m); *R_p_* is the electrical resistance of serial coal pillar specimens (Ω); *S_p_* is the effective cross-sectional area of serial coal pillar specimens (m^2^); *H_p_* is the total height of serial coal pillar specimens (m); *R_u_* and *R_b_* are the electrical resistance of upper and bottom coal pillar specimen (Ω), respectively.

A two-electrode insulation resistance testing system was adopted for measuring the electrical resistance of serial coal pillar specimens. It consisted of resistance testing holders, copper electrodes, insulation boards, a data acquisition instrument and an analysis software. The maximum testing resistance was 1012 Ω, and the measuring deviation was within 1%. The data acquisition frequency was 5 Hz. As shown in Figure 3 and Figure 4, copper electrodes, with dimensions of *a*_2_ × *a*_2_ × *h*_2_ = 200 mm × 200 mm × 1 mm, were fixed symmetrically at both sides of the serial coal pillar specimens. They were sandwiched between insulation boards (*a*_1_ × *a*_1_ × *h*_1_ = 300 mm × 200 mm × 1 mm) and testing coal pillar specimens. Resistance testing holders were connected with upper and bottom copper electrodes, which were also linked with the data acquisition instrument.

#### 2.2.4. Strain Testing System

A strain testing system was utilized to record the axial and lateral strains of serial coal pillar specimens. In this experiment, two pairs of strain gauge rosettes were attached by silastic glue at the central surface of upper and bottom coal pillar specimens, respectively (see Figure 4). Each strain gauge rosette was composed of two strain gauges with a sensitivity of 10^−6^ ε. They were arranged along the horizontal and longitudinal orientations of the specimens. During the fracturing process, the strain gauge rosettes were connected with the data acquisition system by the lead wires. The local strain of serial coal pillar specimens could be recorded continuously.

### 2.3. Experimental Scheme

Three groups of overlapped coal pillar specimens were prepared and tested to obtain the fracturing information under the uniaxial compression condition. Table 2 presents the detail strength of upper and bottom specimens in each group 1–2. The uniaxial compressive strength (UCS) of upper and bottom coal specimens was 10.24 MPa and 7.62 MPa, respectively.

It should be noted that the upper and bottom coal specimen didn’t fail simultaneously due to the different mechanical parameters. That is, two possible conditions may be observed after the local collapsing of upper or bottom coal specimen. One condition was that the upper coal specimen may lose the support of bottom collapsed specimen and become unstable. The other one was that a relatively long distance was formed between the upper loading plate and the bottom coal specimen after the collapse of upper coal specimen. At both conditions, the loading stress could not directly transfer to the non-collapsing coal pillar specimen. The deviation and dropping of AE sensors, copper electrodes and strain gauges, attached on the surface of collapsed coal specimen, may be also observed. Therefore, the data monitoring had to be stopped after the local collapse of upper or bottom coal specimen.

## 3. Experimental Results

In this section, fracturing behaviors of serial coal pillar specimens are presented, which are both described by the mechanical behaviors, AE features, electrical resistivity responses and local strain develops. For different groups of serial coal pillar specimens, the overall evolution of fracturing behaviors was very similar. Therefore, only a group of testing specimens was selected as the representative one. The peak load of the selected serial specimens was very close to the average UCS.

### 3.1. Stress-AE Energy-Electrical Resistivity Response

**Figure 5:**
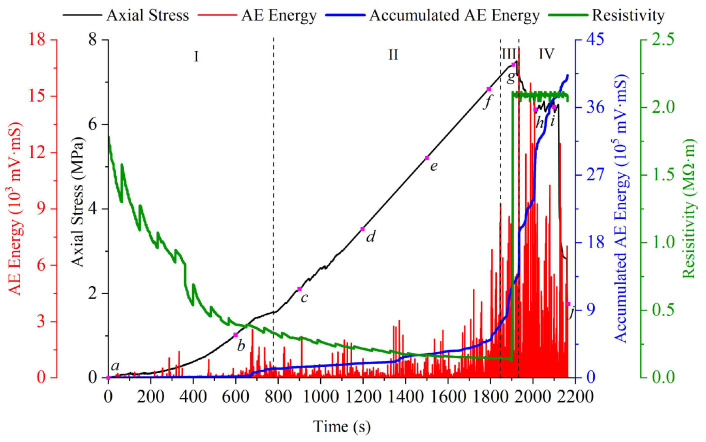
Response of axial stress, AE energy, accumulated AE energy, electrical resistivity for serial coal pillar specimens with different strength.

The curves of axial stress, AE energy, accumulated AE energy and electrical resistivity for the representative specimens are shown in Figure 5. The black curve in Figure 5 presents the response of axial stress. The red curve in Figure 5 shows the evolution of AE energy. The accumulated AE energy is presented as the blue curve in Figure 5. The green curve in Figure 5 exhibits the evolution of electrical resistivity. It should be noted that the electrical resistivity of serial coal pillar specimens in different groups was calculated based on the real-time electrical resistance monitored during the fracturing process.

According to the bearing behaviors, it could be roughly divided into four stages. Stage I was the nonlinear growth stage of bearing capacity. Stage II was the linear growth stage of bearing capacity. Stage III was the yielding growth stage of bearing capacity. Stage IV was the weakening stage of bearing capacity. A thorough description of stress evolution is presented as follows (see Figure 5).

(1) At stage I, axial stress of serial coal pillar specimens was growing with an up-concaved form. Original pores and cracks inside the testing coal pillar specimens were gradually enclosed. That is, the serial coal pillar specimens mainly experienced the compaction deformation at stage I. AE energy was primarily released due to the gradual closure of initial pores and cracks at stage I. A slight fluctuation tendency was detected. It maintained a relatively small value. As a result, a weak growth of accumulated AE energy was observed. The initial electrical resistivity of serial coal pillar specimens was 2.48 MΩ·m before the axial loading, respectively. With the evolution of compaction deformation, original and newborn electrical channels were combined to improve the conductive properties [45]. The electrical resistivity of serial coal pillar specimens was cutting down gradually as a whole. The fluctuation characteristics of electrical resistivity were also revealed at stage I. It could be explained by the heterogeneity of the coal pillar specimen and the uncertainty of initial damage’s expansion direction.

(2) Stage II had the longest evolution time during the entire loading fracturing process. The duration of stage II was approximately 1070s. At this stage, the linear growth of bearing capacity was monitored. That is, elastic deformation was dominant for the serial coal pillar specimens. With the linear growth of bearing capacity, AE behavior of serial coal pillar specimens was activated little by little. The accumulated AE energy presented a gentle smooth increasing tendency at stage II. Moreover, a continuous descending tendency of electrical resistivity was detected at stage II. Due to local damage of conductive channels in the testing specimens, the electrical resistivity was decreasing with a weakening rate. The fluctuation at stage II almost disappeared.

(3) The duration of stage III is shortest for the testing serial specimens. The carrying capacity began to gradually deviate from linear growth, which shown the yield growth trend. The growth rate was weakened slightly at stage III. At the end of this stage, the maximum value of loading capacity developed to 7.50 MPa. Obviously, the limit bearing capacity of serial coal pillar specimens is similar to the UCS of lower coal pillar specimen. The activity of AE behavior became more intense at stage III. Massive AE energies released and rapid growth of accumulated AE energy was observed. The maximum value of AE energy was recorded as soon as the appearance of peak stress. During the stage III, the electrical resistivity maintained a very low level. It still basically presented a decreasing tendency as a whole. At the end of stage III, the electrical resistivity of serial coal pillar specimens increased to 2.09 MΩ·m sharply. It was attributed to the formation and expansion of micro-fracture in the testing coal pillar specimens [43,44]. The dilatation phenomenon was also observed at this moment. The electrical conductive channels then suffered serious damaged and broken [36,45]. As a result, the electrical resistivity presented an accelerated rising tendency.

(4) At stage IV, the carrying capacity of serial coal pillar specimens turned to decrease. At the initial period of stage IV, the weakening rate was relatively slow. The bearing capacity had a weak fluctuation change. Afterwards, a large drop of bearing capacity occurred within a short time. It indicated that the serial coal pillar specimens experienced intense damage and the system stability was seriously destructed. The output of AE energy continued to be intense at stage IV. It was primarily induced by the propagation of macro-cracks. An increasing tendency of accumulated AE energy was monitored at this stage. As shown in Figure 5, the slope of accumulated AE energy, representing the damage rate, was larger enough. It was an illustration about the violent damage. Along with the weakening of bearing capacity, the change of electrical resistivity was relatively weak. This was due to the fact that irreversible damage occurred in the conductive channel of testing specimens after the formation of macro-cracks. It made the overall conductivity challenging to recover.

### 3.2. AE Events Distribution

**Figure 6:**
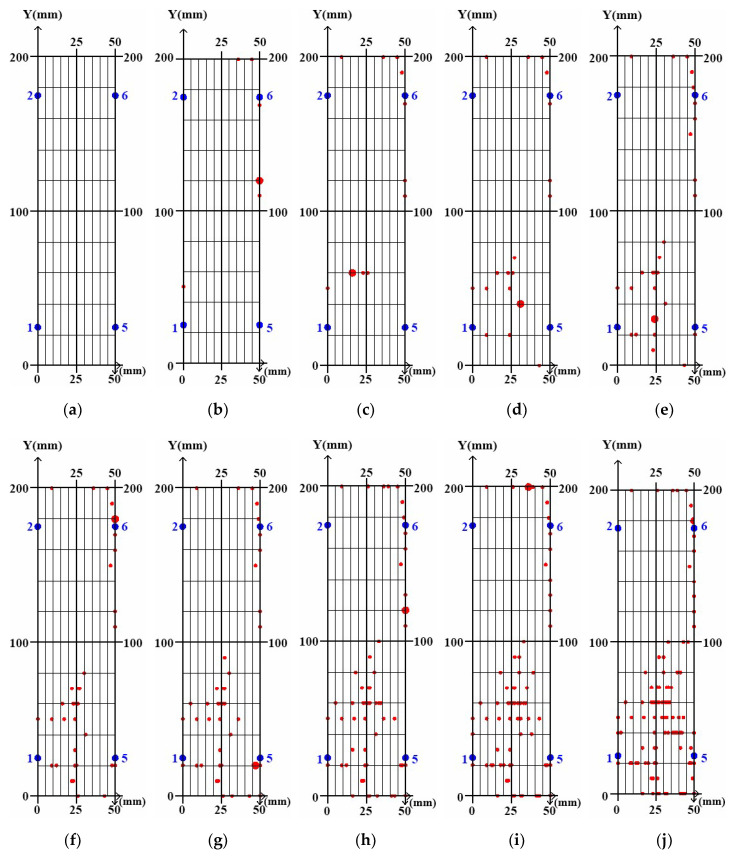
Distribution of AE events for serial coal pillar specimens with different strength. (**a**) 0 s; (**b**) 600 s; (**c**) 900 s; (**d**) 1200 s; (**e**) 1500 s; (**f**) 1800 s; (**g**) 1900 s; (**h**) 2000 s; (**i**) 2100 s; (**j**) 2162 s. Note: The denoted letters shown in the figure was corresponding to those on the stress-time curve in Figure 5.

The stress-AE energy-electrical resistivity response could reflect the overall fracturing behaviors of serial coal pillar specimens. However, it was difficult to explain the local fracturing behaviors of upper specimen or bottom specimen during the loading process. The interaction relationship between the upper specimen or bottom specimen was even more difficult to be revealed. AE features could not only be reflected by the evolution of AE energy quantitatively, but also be revealed by the distribution of AE events qualitatively [40,41]. Therefore, the AE events distribution of serial coal pillar specimens at different loading stages is shown in Figure 6. It is presented to reflect the micro-cracks and internal fractures in the upper specimen or bottom specimen [46,47]. The letters showed in Figure 6 are corresponding to the purple markers and time slices in Figure 5.

Only a sporadic amount of AE events appeared inside the upper coal pillar specimen. No AE events were distributed inside the bottom coal pillar specimen at stage I, as shown in Figure 6a,b. At stage II, earlier AE events appeared inside the left area of bottom coal pillar specimen (lower UCS). The number of AE ruptures inside the specimen increased step by step, which gradually extended to the upper-right area of bottom coal pillar specimen (lower UCS). In this process, the AE events also increased weakly inside the upper coal pillar specimen (higher UCS). The main distribution area was at the right surface area, as shown from Figure 6c–f. At stage III, the AE events distribution density and area increased rapidly inside the bottom coal pillar specimen. It spread with a relatively fast speed. Internal fracturing was still weak for the upper coal pillar specimen (higher UCS), as shown in Figure 6g,h. Almost no new-born AE events appeared [see Figure 6h]. At stage IV, the number and density of AE events scattered inside the bottom coal pillar specimen (lower UCS). The AE events were penetrated throughout the two end surfaces of bottom coal pillar specimen (lower UCS), as shown in Figure 6i,j. This showed that the lower UCS coal pillar specimen became to be failure. At this time, the changes of AE fracturing point in the upper higher UCS coal pillar specimen were still very weak. Only a small number of new rupture points appeared on the right-top edge of the specimen, indicating that the upper coal pillar specimen was hardly fracturing during the entire loading process. After that, the lower coal pillar specimen gradually lost the ability to support the upper coal pillar specimen. This would cause the overall destruction of serial coal pillars.

The above distribution characteristics of AE events showed that: the bottom coal pillar specimen was earlier and more prone to damage. Then, it lost the support of upper coal pillar specimen, which also triggered the destruction of serial coal pillar system. The overall limit bearing capacity of serial coal pillar specimens was 7.50 MPa. It was close to the strength (7.62 MPa) of bottom coal specimen. The above phenomenon was appeared since the UCS of bottom coal pillar specimen was less than that of upper testing specimen. As a result, the systemic fracturing of serial coal pillar specimens was accompanied by the fracturing of lower UCS specimen. It could be concluded that the UCS difference between the upper and bottom specimens was one of the important factors influencing the overall bearing capacity and system stability.

### 3.3. Local Strain Develops

**Figure 7:**
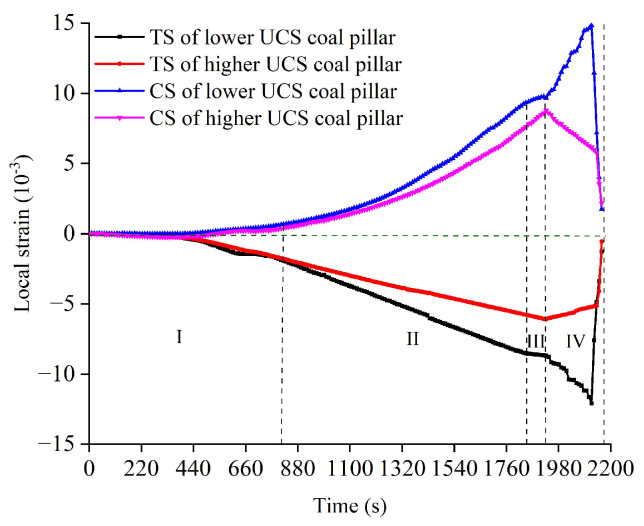
Local strain of serial coal pillar specimens with different UCS.

To make a further explanation about the fracturing of serial coal pillar specimens, the local strain was measured by the strain gauge rosettes. It was widely applied to reflect the local deformation of coal and rock [48,49]. Local strain of testing coal pillar specimens is plotted as Figure 7. The positive value of local strain represents the compressive strain (CS) measured by the longitudinal gauge, while the negative value of local strain means the tensile strain (TS) measured by the horizontal gauge.

(1) At stage I, CS and TS were very small for the testing coal pillar specimens. Nonlinear growth of local strain was observed as a whole.

(2) At stage II, the growth rate of local strain was more rapid for the bottom coal pillar specimen (lower UCS). It revealed that internal fracturing inside the lower UCS coal pillar specimen was more intense. As a result, both the CS and TS of lower UCS coal pillar specimen were larger in value.

(3) At stage III, the stable growth of local strain was monitored for the upper coal pillar specimen (higher UCS). At the peak stress point, the maximum values of CS and TS were captured in the higher UCS coal pillar specimen.

(4) At stage IV, a rapid growth of CS and TS was observed for the bottom coal pillar specimen. Then, the maximum CS and TS values of above specimen were reached. Afterwards, CS and TS began to decline for the upper coal pillar specimen (higher UCS). It was worth to noted that the weakening rate was basically contrary to the growth rate at stage III. That is, the rebounding of deformation was appeared in the corresponding specimens. At the end of stage IV, local strain of higher UCS coal pillar specimen dropped suddenly along with the losing of bearing stress.

In summary, the uniaxial mechanical behaviors were mainly divided into four stages for the serial coal pillar specimens. The AE features, electrical resistivity responses and local strain develops were highly consistent to illustrate the mechanical behaviors. The lower UCS coal pillar specimen was more likely to suffer internal fracturing throughout the loading progress. System fracturing of serial coal pillar specimens was appeared along with the collapse of lower UCS coal pillar specimen. The limit bearing capacity of serial coal pillar specimens with different UCS was almost equal to the strength of lower UCS coal pillar specimen.

## 4. Numerical Simulation

To make a further demonstration about experimental results’ accuracy, the numerical fracturing behaviors were investigated by particle flow code (PFC) for the serial coal pillars with different strength.

### 4.1. Establishment of Numerical Model

**Figure 8:**
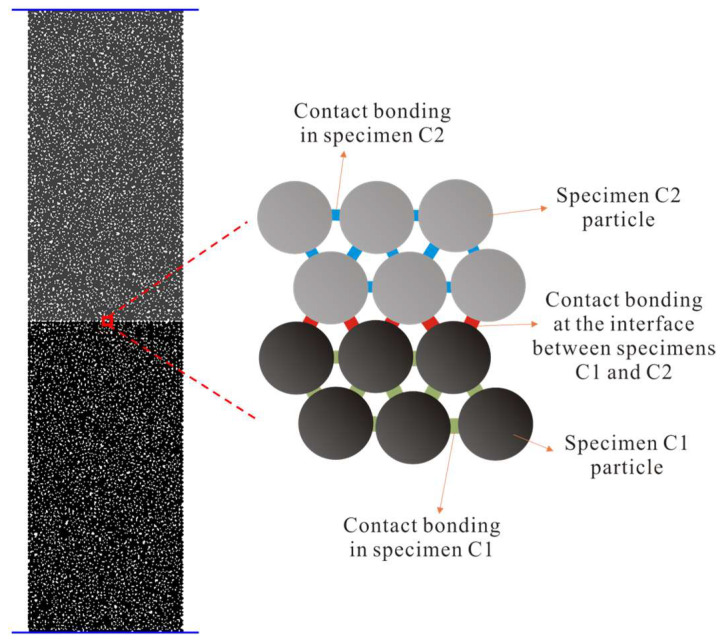
Numerical model of serial coal pillar specimen.

PFC is a discrete element numerical simulation software that is widely used in the study of the mechanical behavior of coal and rock. The numerical model of serial coal pillar specimens was shown in Figure 8. In accordance with laboratory tests, a model of the specimen was constructed with a diameter of 50 mm and a height of 200 mm, using the same scale as the experimental specimen. The particle size was set to 0.5–0.75 mm. The specimens constituted 7217 particles. The model consisted of bottom coal pillar specimen C1 and upper coal pillar specimen C2. Parallel bonding model was used for the contact model of the specimens to reflect the mechanical properties. Table 3 showed the meso-parameters used in the PFC model for serial coal pillar specimens in this research.

**Figure 9:**
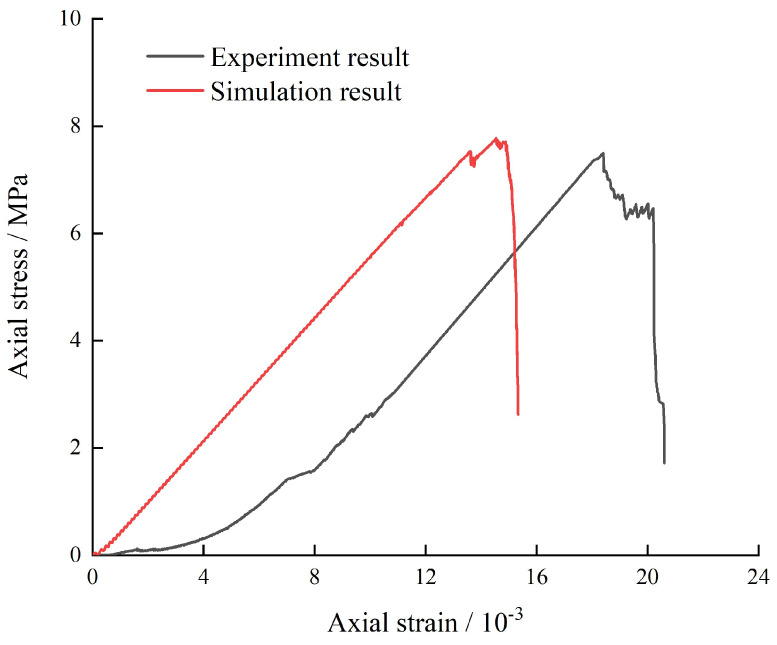
Comparison between the numerical simulations and experimental results.

Three types of contact bonding models were applied to simulate the serial coal pillar specimens: the contact bonding in specimen C1, the contact bonding in specimen C2, and the contact bonding at the interface of C1 and C2. Figure 9 shows the comparison of stress-strain curves obtained from experimental and numerical serial coal pillar specimens under the uniaxial compression. It could be seen that the numerical results agreed well with the experimental results.

### 4.2. Stress Distribution Pattern

**Figure 10:**
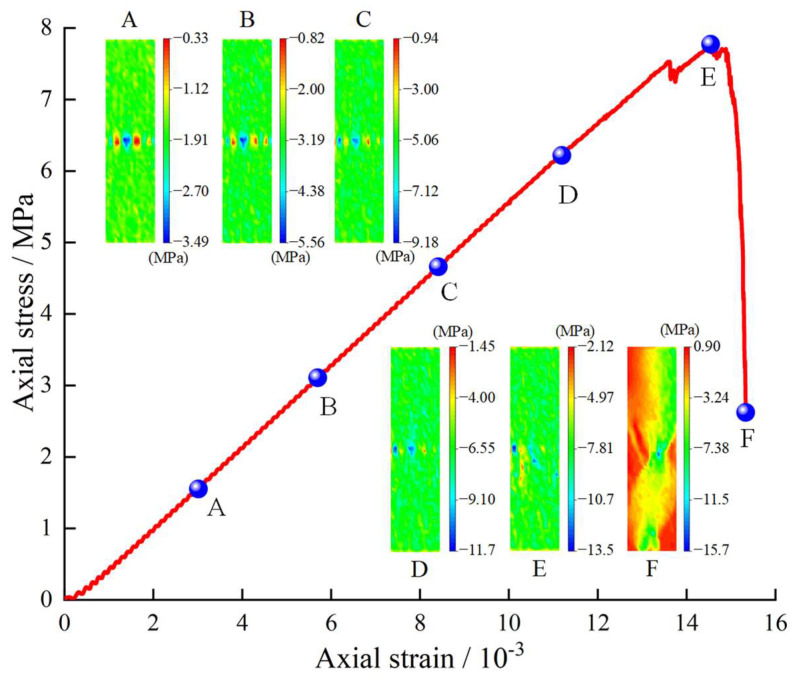
Numerical stress evolution pattern of serial coal pillar specimens with different strength.

To obtain the stress evolution pattern, 1200 measurement circles were regularly arranged inside the serial specimens during the loading process. The measurement circles had a certain amount of overlapping to ensure the particles in the range were monitored completely. In the loading direction, the numerical stress evolution pattern of serial coal pillar specimens was shown in Figure 10. Six representative stress state were presented Figure 10. The stress of point A state was 20% of maximum bearing capacity. The stress of point B state was 40% of maximum bearing capacity. The stress of point C state was 60% of maximum bearing capacity. The stress of point D state was 80% of maximum bearing capacity. The stress of point E state was equal to the maximum bearing capacity. The stress of point F state was 35% of maximum bearing capacity after the peak stress. It should be noted that the positive stress values indicated the tensile stress, while the negative stress values meant the compressive stress.

**Figure 11:**
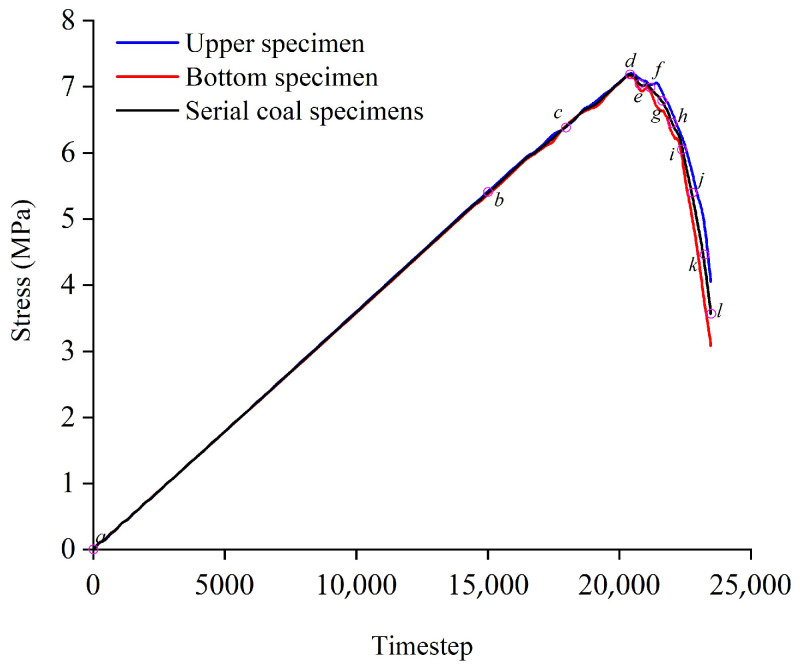
Quantitative relationship between the stress and timestep of serial coal pillar specimens.

It could be seen from Figure 10 that the compressive stress existed within the serial coal pillar specimens before the peak stress. At point A, the compressive stress was relatively low and the stress concentrations were found on the interface of upper-bottom specimens. From point B to point E, the stress concentration degree decreased. With the growth of loading, the stress became progressively uniform within the serial specimens. At point F, the non-uniform tensile stress zone appeared inside the serial coal pillar specimens. It indicated that the tensile stress was accompanied with the post-peak stage. However, no significant differences were observed in the stress distribution between the upper and lower coal pillar specimens. That is, the serial coal pillar specimens had a balanced bearing characteristic, which could also be confirmed by the following stress data (see Figure 11).

**Figure 12:**
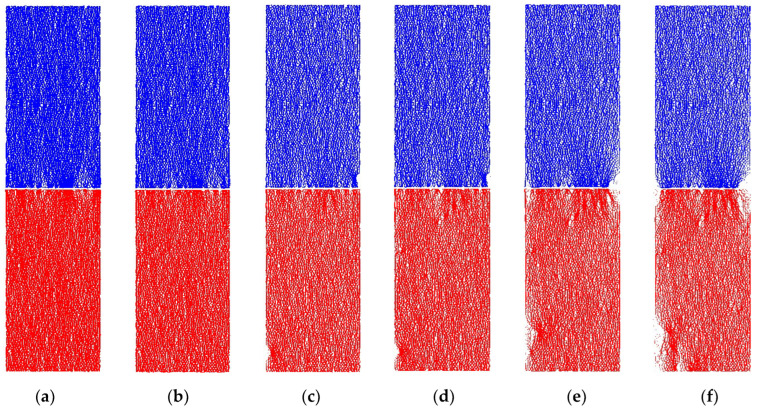

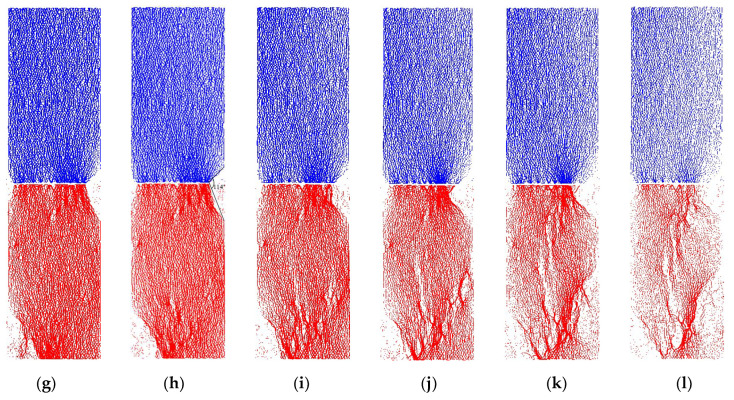
Evolution of contact-force chain serial coal pillar specimens. (**a**) 0; (**b**) 15,000; (**c**) 18,000; (**d**) 20,400; (**e**) 20,800; (**f**) 21,200; (**g**) 21,600; (**h**) 22,000; (**i**) 22,400; (**j**) 22,800; (**k**) 23,200; (**l**) 23,487. Note: The denoted letters shown in the figure was corresponding to those on the stress-time curve in Figure 11.

Figure 11 presented the quantitative stress-time relationship of upper testing specimen, bottom testing specimen and serial coal pillar specimens, which were expressed as the blue curve, red curve and black curve. Figure 12 represents the contact force chain distribution of serial coal pillar specimens during the loading process. The step in Figure 12 was corresponding to the purple time circle in Figure 11.

Obviously, the carrying capacity of the upper testing specimen and bottom testing specimen was basically equal in value. The overall bearing capacity of the series coal pillar system was also equal to the independent carrying capacity of testing specimen. However, there were many differences in the contact force chain distribution between the upper coal specimen and bottom specimen. It could be seen from Figure 12 that the rupture, bifurcation, extension and penetration of contact force chain mainly occurred in the bottom coal pillar specimen (lower UCS). When the macro-contact force chain fracture zone appeared in the bottom coal pillar specimen, local instability was caused. It would lose the support for the upper coal pillar specimen (higher UCS). Eventually, the systemic fracturing of serial coal pillar specimens was induced.

### 4.3. Crack Evolution

**Figure 13:**
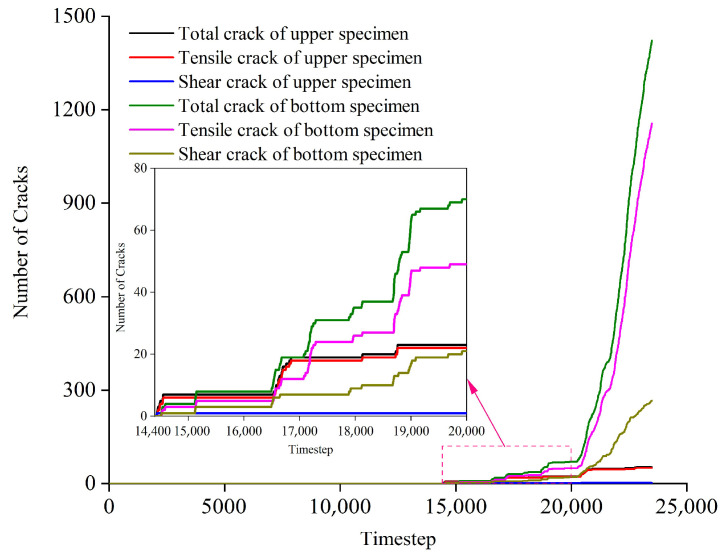
Evolution curve of crack number of serial coal pillar specimens.

**Figure 14:**
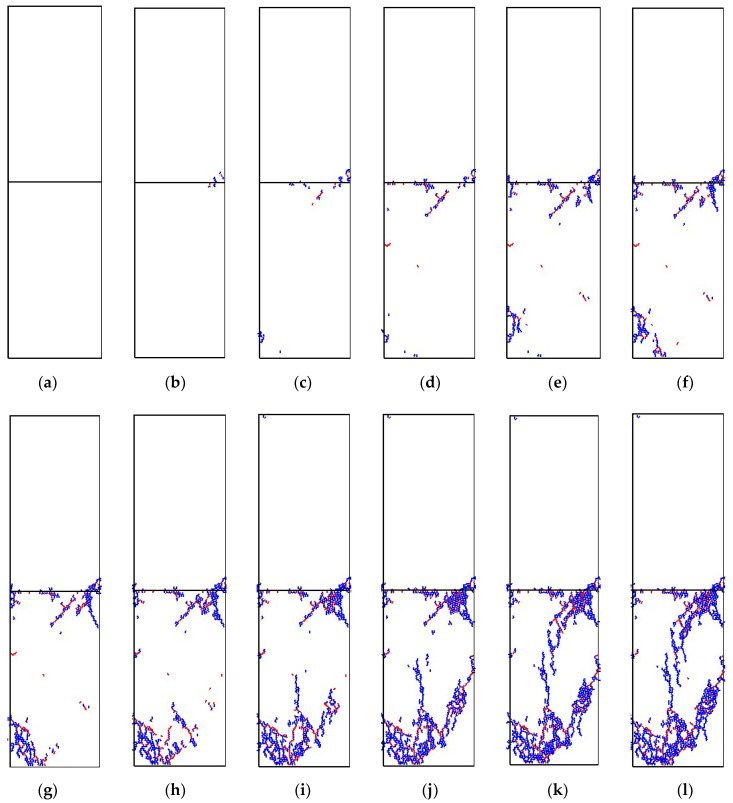
Evolution of crack form and location of serial coal pillar specimens. (**a**) 0; (**b**) 15,000; (**c**) 18,000; (**d**) 20,400; (**e**) 20,800; (**f**) 21,200; (**g**) 21,600; (**h**) 22,000; (**i**) 22,400; (**j**) 22,800; (**k**) 23,200; (**l**) 23,487.

From a qualitative and quantitative perspective, the crack number, form, and location of serial coal pillar specimens were investigated in the following section. Figure 13 and Table 4 showed the cracks number evolution of upper specimen, bottom specimen and serial coal pillar specimens. Figure 14 represented the cracks form and location of upper specimen, bottom specimen and serial coal pillar specimens at different loading timestep. It should be pointed out that the blue lines in the Figure 14 indicated the tensile cracks, and the red lines meant the shear cracks.

When the loading timestep was smaller than 14,400, it could be seen that no micro-cracks were generated inside the serial coal pillar specimens. Micro-cracks firstly appeared in both the upper coal specimen and bottom coal specimen when the timestep ranged from 14,400 to 15,000, which were mostly distributed near the interface, as shown in Figure 14a,b. When the loading timestep was 15,000, the number of tensile cracks and shear cracks was 6 and 1 inside the upper coal specimen, respectively. For the bottom coal specimen, the number of tensile cracks and shear cracks was 3 and 1.

When the loading timestep was 15,000~20,400, the number of micro-cracks in the upper specimen and bottom specimen increased slowly, but it was mainly sprouted and expanded in the bottom specimen, as shown in Figure 14. Specifically, the number of tensile cracks in the upper specimen grew from 6 to 22, and the number of shear cracks increased from 1 to 2. In the bottom specimen, the tensile cracks number increased to 58 from 3, while the shear crack number grew to 31 from 1. Moreover, the micro-cracks were mainly distributed in the lower-left corner and upper-right corner area in the bottom specimen, as shown in Figure 14b–d.

When the loading timestep was 20,400~22,800, the micro-cracks in the upper specimen still grew slowly. The number of tensile cracks and shear cracks increased to 50 and 3, respectively. However, the micro-cracks in the bottom specimen showed a rapid growth trend, as shown in Figure 13 and Table 4. The number of tensile cracks in the bottom specimen increased from 58 to 869, and the number of shear cracks rose from 31 to 220. Meanwhile, the tensile and shear cracks were still mainly distributed lower-left corner and upper-right corner area in the bottom specimen. The distribution area also gradually increased. That is, the micro-cracks in the above two distribution areas gradually extended and gathered. However, the two areas did not penetrate each other. In the end, a W shaped cracks presented in the lower-left corner, and a triangular shaped cracks gathered in the upper-right corner, as shown in Figure 14d–j.

When the loading timestep was 22,800~23,487, the number of micro-cracks remained unchanged inside the upper specimen, as shown in Figure 13 and Table 4. The micro-cracks continued to increase sharply in the bottom specimen. The number of tensile cracks and shear cracks increased to 1156 and 266, respectively. It should be pointed out that the micro-cracks gradually expanded at the lower-left corner and upper-right corner. As a result, the cracks were penetrated throughout each other and formed several macro cracks, as shown in Figure 14j–l. It also indicated the macro-failure of bottom specimen, which lost the support to the upper specimen. It was believed that the system stability could not be maintained for the serial coal pillar stability.

In summarize, the germination, extension and penetration of micro-cracks occurred mainly in the specimen with lower UCS. Local instability appeared of lower UCS specimen when the macro-cracks generated. Then, the chain fracturing of higher UCS specimen was accompanied. Then, the overall fracturing of serial coal pillar specimens was also induced.

### 4.4. Local Displacement

**Figure 15:**
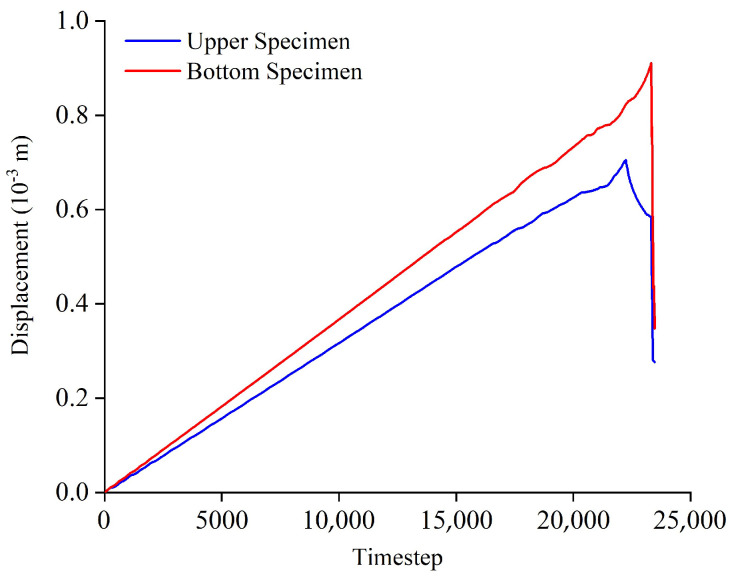
Local displacement evolution of upper and bottom specimens.

Figure 15 represented locally the displacement-timestep relationship of upper and bottom specimens. It can be seen from Figure 15 that: the vertical displacement of bottom specimen was always larger than that of upper specimen during the loading process. There were large differences in the displacement generated by the upper and the bottom specimen. That is, the serial coal pillar specimens showed obvious non-balanced deformation characteristics under the axial loading condition.

## 5. Discussion

The related numerical results and experimental results were consistent as a whole. The common conclusion could be obtained that the unbalanced deformation characteristics of serial coal pillar specimens was presented. The balanced bearing characteristics were also exhibited. In order to make a deeper illustration about the related phenomenon, a schematic model of serial coal pillar specimens was established in the following section. Then, the progressive fracturing mechanism of serial coal pillar specimens with different UCS was investigated.

### 5.1. Schematic Model of Serial Coal Pillar Specimens

**Figure 16:**
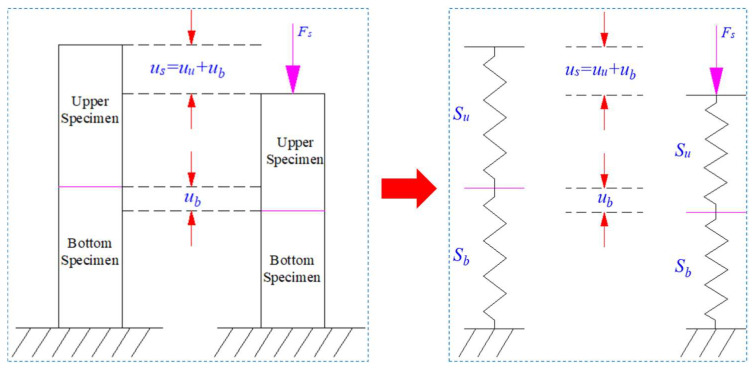
Schematic model of serial coal pillar specimens before and after the uniaxial loading.

Figure 16 showed the schematic model of serial coal pillar specimens with different strength. In the one-degree-of-freedom system, two nonlinear and inelastic coal pillar specimens were connected in serial [20]. Furtherly, the upper specimen and bottom specimen were simplified to two springs, which was represented by *S_u_* and *S_b_*, respectively. That is, the serial coal pillar specimens were regarded to be composed by two interconnected springs. Both of them were possessed with the softening and hardening characteristics. For the serial coal pillar specimen model, the UCS of upper pillar specimen was more than the bottom one, while the elastic modulus was same in value.

For the upper and bottom coal pillar specimen, the relationship between the bearing load and displacement could be expressed by Equation (3) [14,21].

(3) $$\{\begin{array}{c} F_{u}=f_{u}(u_{u})\\ F_{b}=f_{b}(u_{b}) \end{array}$$

where *F_u_* and *F_b_* meant the bearing load of upper and bottom coal pillar specimen, respectively. *u_u_* and *u_b_* represented the vertical displacement of upper and bottom coal pillar specimen, respectively.

When the interaction between upper and bottom pillar specimens was regarded to be quasi-static, the constitutive relation of serial coal pillar system could be described by Equation (4).

(4) $$\{\begin{array}{c} F_{s}=F_{u}=F_{b}\;\\ u_{s}=u_{u}+u_{b}\; \end{array}$$

where *F_s_* was the global bearing load of serial coal pillar specimens. *u_s_* was the total displacement of serial coal pillar specimens.

Furthermore, the relationship between bearing load and displacement for serial coal pillar specimens could be derived as the Equation (5) [14]:

(5) $$F_{s}=f_{s}^{\prime }(u_{s})\cdot u_{s}=\frac{f_{u}^{\prime }(u_{u})\cdot f_{b}^{\prime }(u_{b})}{f_{u}^{\prime }(u_{u})+f_{b}^{\prime }(u_{b})}u_{s}\;$$

where $f_{s}^{\prime }(u_{s})$ was the secant modulus of serial coal pillar specimens, $f_{u}^{\prime }(u_{u})$ and $f_{b}^{\prime }(u_{b})$ were the secant modulus of upper and bottom coal pillars, respectively.

### 5.2. Fracturing Evolution Analysis

**Figure 17:**
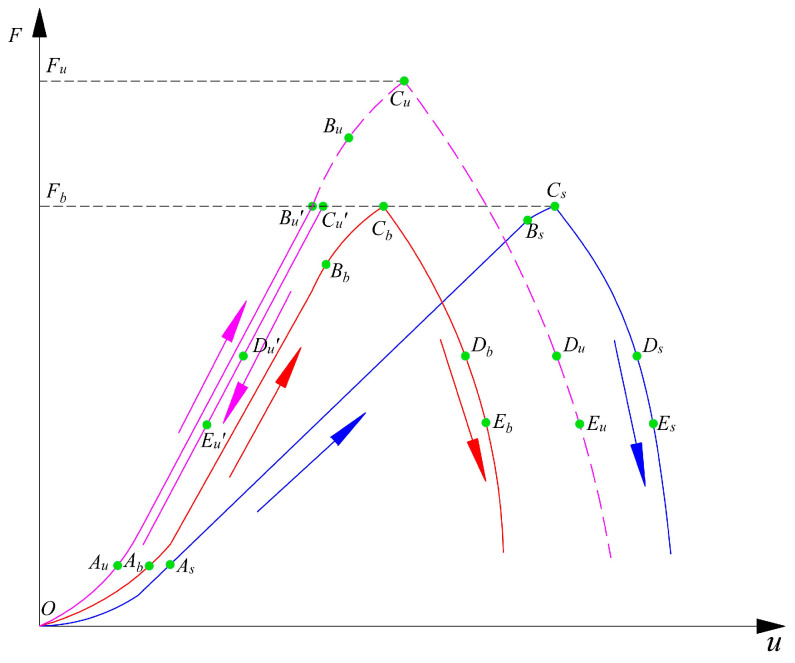
Relationship of load-displacement for serial coal pillar specimens (different strength).

According to the fracturing behaviors of serial coal pillar specimens, the deformation of upper and bottom pillar specimens was not always coordinated during the loading process. Figure 17 presented the schematic relationship of load-vertical displacement for serial coal pillar specimens (different strength) during the whole loading process. *F_u_* and *F_b_* mean the limit bearing load of upper and bottom coal specimen, respectively. The load-displacement relation of lower UCS coal pillar specimen was expressed by the red curve, while it was plotted by the purple curve for the higher UCS coal pillar specimen. For the dashed purple curve in Figure 17, it stood for the hypothetical variation path of load-displacement for higher UCS coal pillar specimen when the bearing load was more than *F_b_.* Without the effect of lower UCS coal pillar specimen, it would experience the strain hardening and softening stage when the stress was more than *F_b_.* The solid purple curve in Figure 17 represented the actual evolution path of higher UCS coal pillar specimen during the loading process. Obviously, there was a significant rebound deformation when the stress was more than *F_b_.* Moreover, the blue curve represented the global load-displacement relation of serial coal pillar specimens.

From Figure 17, the unbalanced deformation characteristics were illustrated once again for the serial coal pillar specimens. Therefore, the key deformation element was defined to reveal the unbalanced deformation characteristics in this study. It was determined by comparing the values of *u_u_* and *u_b_*. Both the testing coal pillar specimens were regarded as the key deformation elements when *u_u_* = *u_b_*. The upper coal pillar specimen, generating more deformation than the bottom one, was considered as the key deformation element. Contrarily, the key deformation element was the bottom coal pillar specimen when *u_u_* < *u_b_*.

Specifically, the fracturing evolution of serial coal pillar specimens was analyzed. The deformation of upper and bottom testing specimens at the initial loading period was basically coordinated. Both the upper and bottom coal pillar specimens were regarded as the key deformation elements. Then, the bottom coal pillar specimen (lower UCS) turned to be the key deformation element. Owing to the lower strength, the axial stress was earlier to reach the limit bearing load of bottom coal pillar specimen. As a result, the fracturing evolution of lower UCS coal pillar specimen was presented as the solid path: *O* → *A_b_* → *B_b_* → *C_b_* → *D_b_’* → *E_b_*. At that same time, the higher UCS coal pillar specimen was still at the elastic deformation period. Afterward, the bearing load of lower UCS coal pillar specimen was gradually weakened. It would induce the elastic rebounding deformation of higher UCS coal pillar specimen. Consequently, the fracturing evolution of higher UCS coal pillar specimen was presented as the solid path: *O* → *A_u_* → *B_u_’* → *C_u_’* → *D_u_’* → *E_u_’*.

### 5.3. Progressive Fracturing Mechanism

The fracturing of serial coal pillar specimens was progressive. Table 5 summarized the key deformation element evolution and unbalanced bearing characteristics at different the loading stages. The following results could be obtained:

The $f_{u}^{\prime }(u_{u})$ and $f_{b}^{\prime }(u_{b})$ were almost equal at stage I. The values were increasing with an up-concaved form. Then, a linear growth of $f_{u}^{\prime }(u_{u})$ and $f_{b}^{\prime }(u_{b})$ was appeared at stage II. Continuously, the $f_{u}^{\prime }(u_{u})$ was the same as $f_{b}^{\prime }(u_{b})$ in value. At stage III, the elastic deformation was still observed in the upper coal pillar specimen (higher UCS). The $f_{u}^{\prime }(u_{u})$ was equal to the value at stage II. For the $f_{b}^{\prime }(u_{b})$, it began to decreasing gradually due to the axial stress was more than the yield strength of bottom coal pillar specimen. After the peak stress of serial coal pillar specimens, $f_{u}^{\prime }(u_{u})$ was continuously more than 0 due to the elastic rebounding deformation. $f_{b}^{\prime }(u_{b})$ turned to be less than 0 due to the weakening of bearing stress. When the $\left|f_{b}^{\prime }(u_{b})\right|$ was less than $f_{u}^{\prime }(u_{u})$, the total value of $f_{u}^{\prime }(u_{u})$ and $f_{b}^{\prime }(u_{b})$ was more than 0. At the point of *D_u_*′, $f_{u}^{\prime }(u_{u})+f_{b}^{\prime }(u_{b})=0$ was obtained since the $\left|f_{b}^{\prime }(u_{b})\right|$ was equal to $f_{u}^{\prime }(u_{u})$ in value (see points *D_b_*, *D_u_*′ or *D_s_* in Figure 17). At that time, the overall bearing load (*F_s_*) was weakened towards 0. This was evidence of system fracturing for serial coal pillar specimens.

Above all, coal pillar specimen with lower UCS in the serial system was the key deformation element during the progressive fracturing process, especially at the later loading stages. Internal fracturing of lower UCS coal pillar specimen could induce energy release and rebounding deformation of higher UCS coal pillar specimen. It would accelerate the fracturing of higher UCS coal pillar specimen. That is, the lower UCS coal pillar could be determined as the key pillar. The local instability of key coal pillar could furtherly induce the overall chain fracturing of serial pillar system. The critical condition of system fracturing was $f_{u}^{\prime }(u_{u})+f_{b}^{\prime }(u_{b})=0$ for the serial coal pillar specimens with different UCS.

## 6. Conclusions

In this paper, uniaxial compression tests of serial coal pillar specimens with different strengths were conducted. The “stress-acoustic-electric” response characteristics of specimens during the failure process were analyzed. The PFC2D software was used to analyze the force chain evolution and crack development characteristics. The main conclusions are as follows:

(1) The bearing stress of serial coal pillar specimens are mainly divided into four stages during the loading process: nonlinear growth stage of bearing capacity, linear growth stage of bearing capacity, yielding growth stage of bearing capacity, weakening stage of bearing capacity. The AE features, electrical resistivity responses, strain develops, force chain distributions, cracks evolutions, and local displacement are highly consistent to illustrate the fracturing behaviors.

(2) The lower UCS coal pillar specimen is more likely to suffer internal damage throughout the loading progress. System fracturing of serial coal pillar specimens is appeared along with the collapse of lower UCS coal pillar specimen. The limit bearing capacity of serial coal pillar specimens is almost equal to the strength of lower UCS coal pillar specimen.

(3) Due to the differences of UCS, the unbalanced deformation characteristics of serial coal pillar specimens are presented. The balanced bearing characteristics are also exhibited. The key deformation element is defined to reflect the unbalanced deformation characteristics. The evolution of key deformation element is the rooted reason to account for the overall fracturing mechanism of serial coal pillar specimens.

(4) For the serial coal pillar specimens with different UCS, both the lower and higher UCS coal pillar specimens are the initial key deformation element. It turned out to be the lower UCS coal pillar specimen at the later loading stages. The limit bearing capacity of lower UCS coal pillar specimen reached earlier, while higher UCS coal pillar specimen is still at the elastic deformation stage. Rebounding deformation of higher UCS coal pillar specimen occurred after the peak stress. It would accelerate the system fracturing of lower UCS coal pillar specimen. The limit carrying capacity is almost equal to the strength of lower UCS coal pillar specimen.

(5) The critical condition of system fracturing is $f_{u}^{\prime }(u_{u})+f_{b}^{\prime }(u_{b})=0$ for serial coal pillar specimens with different strength, which is expect to predict the system fracturing of serial pillar in the underground coal mining.

## Figures and Tables

**Table 1:** Physical and mechanical parameters of coal specimens.

Type	Diameter/mm	Height/mm	Mass/g	Density/ (g/cm^3^)	Initial Resistivity/ (MΩ·m)	Peak Force/KN	Uniaxial Compressive Strength/MPa
I	49.94	99.93	253.21	1.29359	2.45	20.11	10.24
II	49.96	100.15	187.61	0.95559	0.96	14.96	7.62

**Table 2:** Parameters of serial coal pillar specimens with different strength.

Serial Coal Pillar Specimen	Pillar Type	UCS/MPa
Upper Coal Specimen	I	10.24
Bottom Coal Specimen	II	7.62

**Table 3:** Meso-Parameters of serial coal pillar specimens with different strength.

Meso-Mechanical Parameters	Values
Particle minimum radius, (mm)	0.5
Particle radius ratio	1.5
The density of the particle, (kg/m^3^)	2500
The damp of the particle	0.7
Friction coefficient of particles	0.577
Bond modulus of specimen C1, (GPa)	0.43
Bond modulus of specimen C2, (GPa)	0.4
Bond tensile strength of specimen C1, (MPa)	3.75
Bond tensile strength of specimen C2, (MPa)	4.7
Bond cohesion of specimen C1, (MPa)	3.75
Bond cohesion of specimen C2, (MPa)	4.7

**Table 4:** Evolution of crack number of serial coal pillar specimens at the remarkable timestep.

Timestep	Upper Specimen			Lower Specimen			Serial Coal Pillar Specimens		
	Total Cracks	Tensile Cracks	Shear Cracks	Total Cracks	Tensile Cracks	Shear Cracks	Total Cracks	Tensile Cracks	Shear Cracks
0	0	0	0	0	0	0	0	0	0
15,000	7	6	1	4	3	1	11	9	2
18,000	19	18	1	35	26	9	54	44	10
20,400	24	22	2	89	58	31	113	80	33
20,800	46	43	3	205	150	55	251	193	58
21,200	48	45	3	290	219	71	338	264	74
21,600	48	45	3	391	299	92	439	344	95
22,000	48	45	3	578	434	144	626	479	147
22,400	50	47	3	835	645	190	885	692	193
22,800	53	50	3	1089	869	220	1142	919	223
23,200	53	50	3	1309	1057	252	1362	1107	255
23,487	53	50	3	1422	1156	266	1457	1206	269

**Table 5:** Progressive fracturing evolution of serial coal pillar specimens (different strength).

Stage	Descriptions	Unbalanced Deformation Characteristics	Key Deformation Element
I	Stage I was from *O* to *A_s_* for serial coal pillar specimens	$\left(1)\;f_{u}^{\prime }(u_{u}\right)$$\;\mathrm{and}\;f_{b}^{\prime }(u_{b})$ were 0 at the beginning of stage I. $(2)\;f_{u}^{\prime }(u_{u})>0$. $(3)\;f_{b}^{\prime }(u_{b})>0$. $\left(4)\;f_{u}^{\prime }(u_{u}\right)$$\;\mathrm{and}\;f_{b}^{\prime }(u_{b})$ were both increasing nonlinearly. $(5)\;f_{u}^{\prime }(u_{u})+f_{b}^{\prime }(u_{b})>0$. $(6)\;u_{u}\approx u_{b}$.	Upper and bottom coal pillar specimens
II	Stage II was from *A_s_* to *B_s_* for serial coal pillar specimens	$(1)\;f_{u}^{\prime }(u_{u})>0$. $(2)\;f_{b}^{\prime }(u_{b})>0$. $\left(3)\;f_{u}^{\prime }(u_{u}\right)$$\;\mathrm{and}\;f_{b}^{\prime }(u_{b})$ were both constant. $\left(4)\;f_{u}^{\prime }(u_{u})=f_{b}^{\prime }(u_{b}\right)$. $(5)\;f_{u}^{\prime }(u_{u})+f_{b}^{\prime }(u_{b})=2f_{u}^{\prime }(u_{u})>0$. $(6)\;u_{u}\approx u_{b}$.	Upper and bottom coal pillar specimens
III	Stage III was from *B_s_* to *C_s_* for serial coal pillar specimens	$(1)\;f_{u}^{\prime }(u_{u})>0$. $(2)\;f_{b}^{\prime }(u_{b})>0$. $\left(3)\;f_{u}^{\prime }(u_{u}\right)$ was basically equal to the one at stage II. $\left(4)\;f_{b}^{\prime }(u_{b}\right)$ was decreasing gradually. $\left(5)\;f_{b}^{\prime }(u_{b}\right)$ declined to 0 when the peak stress of serial coal pillar specimens was observed. $(6)\;f_{u}^{\prime }(u_{u})+f_{b}^{\prime }(u_{b})>0$. $(7)\;u_{u}<u_{b}$.	Bottom coal pillar specimen with lower UCS
IV	Stage IV was from *C_s_* to *E_s_* for serial coal pillar specimens	$(1)\;f_{u}^{\prime }(u_{u})>0$. $(2)\;f_{b}^{\prime }(u_{b})<0$. $(3)\;\left|f_{b}^{\prime }(u_{b})\right|$$\;\mathrm{before}\;\mathrm{the}\;\mathrm{point}\;\mathrm{of}\;D_{b}^{\prime }\;\mathrm{was}\;\mathrm{less}\;\mathrm{than}\;f_{u}^{\prime }(u_{u})$$\;\mathrm{before}\;\mathrm{the}\;\mathrm{point}\;\mathrm{of}\;D_{u}.\;\mathrm{The}\;\mathrm{value}\;\mathrm{of}\;f_{u}^{\prime }(u_{u})+f_{b}^{\prime }(u_{b})$ was more than 0. $\left(4)\;\left|f_{b}^{\prime }(u_{b})\right|=f_{u}^{\prime }(u_{u}\right)$$\;\mathrm{and}\;f_{u}^{\prime }(u_{u})+f_{b}^{\prime }(u_{b})=0$ were met at the points of *D_b_’* and *D_u_*. $(5)\;\left|f_{b}^{\prime }(u_{b})\right|$$\;\mathrm{after}\;\mathrm{the}\;\mathrm{point}\;\mathrm{of}\;D_{b}^{\prime }\;\mathrm{were}\;\mathrm{more}\;\mathrm{than}f_{u}^{\prime }(u_{u})$$\;\mathrm{before}\;\mathrm{the}\;\mathrm{point}\;\mathrm{of}\;D_{u}.\;f_{u}^{\prime }(u_{u})+f_{b}^{\prime }(u_{b})<0$. $(6)\;u_{u}<u_{b}$.	Bottom coal pillar specimen with lower UCS

## Data Availability

The experimental data used to support the findings of this study are included within the article.

## List of Symbols

*UCS*	Uniaxial compressive strength
*E*	Elastic modulus
*AE*	Acoustic emission
*H*	Height of single coal pillar specimen
*r*	Radius of coal pillar specimen
*a*	Length and width of loading plate
*h*	Height of loading plate
*a*_1_	Length and width of insulation board
*a*_2_	Length and width of copper electrode
*U*	Voltage of insulation resistance testing system
*h*_1_	Height of insulation board
*h*_2_	Height of copper electrode
*ρ*	Electrical resistivity of a single coal pillar specimen
*R*	Electrical resistance of a single coal pillar specimen
*S*	Effective cross-sectional area of a single coal pillar specimen
*ρ_p_*	Electrical resistivity of serial coal pillar specimens
*R_p_*	Electrical resistance of serial coal pillar specimens
*S_p_*	Effective cross-sectional area of serial coal pillar specimens
*H_p_*	Total height of serial coal pillar specimens
*R_u_, R_b_*	Electrical resistance of upper and bottom coal pillar specimen
CS	Compressive strain
TS	Tensile strain
C1	Bottom coal pillar specimen
C2	Upper coal pillar specimen
*S_u_, S_b_*	Two springs simplified for the top and bottom specimens
*F_u_, F_b_*	Bearing load of upper and bottom coal pillar specimen
*u_u_, u_b_*	Vertical displacement of upper and bottom coal pillar specimen
*F_s_*	Global bearing load of serial coal pillar specimens
*u_s_*	Total displacement of serial coal pillar specimens
*f’_s_(u_s_)*	Secant modulus of serial coal pillar specimens
*f’_u_(u_u_), f’_b_(u_b_)*	Secant modulus of upper and bottom coal pillars
